# Medical Management of Cesarean Scar Ectopic Pregnancy: A Unique Approach

**DOI:** 10.7759/cureus.55481

**Published:** 2024-03-04

**Authors:** Pankaj Salvi, Vidya Gaikwad, Ayushi Bhadoriya, Sanjay Ponde

**Affiliations:** 1 Department of Obstetrics and Gynaecology, Dr. D. Y. Patil Medical College, Hospital & Research Centre, Pune, IND

**Keywords:** gynecology and obstetrics, mifepristone, methotrexate ectopic, medical management, scar ectopic pregnancy

## Abstract

One of the rarest types of ectopic pregnancy, with an incidence of 1:1,800, is cesarean scar ectopic pregnancy. Here, we report the case of a 28-year-old woman who had undergone two previous cesarean sections. She arrived at our labor room with per vaginal spotting and abdominal pain with an ultrasound that revealed a cesarean scar ectopic pregnancy. The initial beta-human chorionic gonadotropin (β-hCG) value upon admission was 27,133 mIU/mL. Her ultrasound findings were confirmed with magnetic resonance imaging. Opting for combined medical management, we successfully treated her using systemic methotrexate and mifepristone, avoiding surgical intervention despite high β-hCG values. There is currently no established standardized treatment for cesarean scar ectopic pregnancies, and we feel that treatment must be tailored to every patient’s individual needs. Our experience suggests that combining mifepristone and systemic methotrexate can be an effective approach with better curative effects, emphasizing the need for further research.

## Introduction

Cesarean scar ectopic pregnancy (CSEP) by definition is a pregnancy in a prior cesarean scar. There has been a substantial increase in the incidence of CSEP due to an increase in the number of cesarean sections worldwide. It is associated with severe maternal morbidities such as massive hemorrhage, uterine rupture, or live births complicated by placenta accrete spectrum, hemorrhage, and/or risk of cesarean hysterectomy.

Common symptoms of this condition include lower abdominal pain and vaginal bleeding, while, in some cases, individuals may even be asymptomatic during the first trimester. The preferred diagnostic method is transvaginal ultrasound, often complemented by a transabdominal scan to obtain a comprehensive view. In situations where the diagnosis is uncertain, magnetic resonance imaging (MRI) can be employed to either confirm or dismiss the presence of the condition. Prompt and precise diagnosis, coupled with timely intervention, can prevent potential pregnancy complications, including hemorrhage and uterine rupture. The choice of treatment depends on the specific presentation of the case. Women may undergo expectant management, receive medical intervention using methotrexate, or opt for surgical procedures. To date, definite guidelines for the management of CSEP have not yet been defined.

## Case presentation

**Figure 1:**
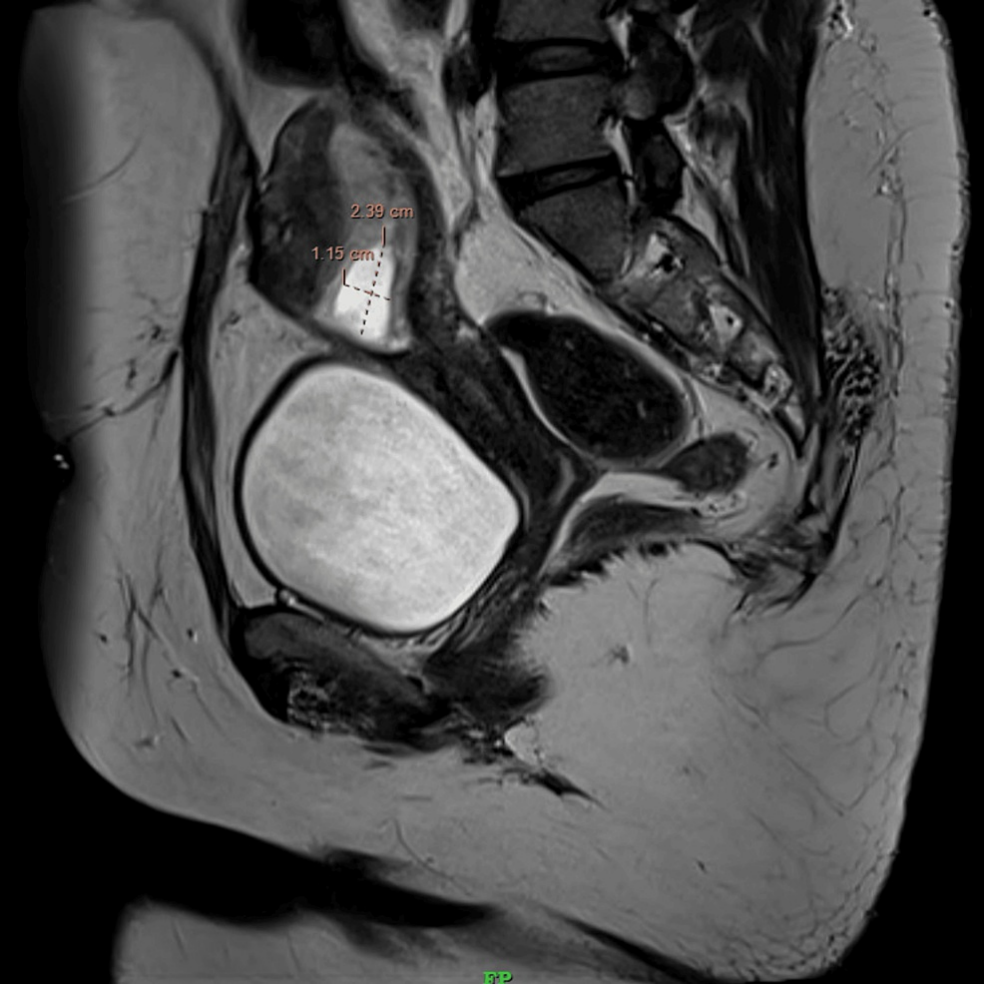
T2-weighted sagittal magnetic resonance imaging showing the gestational sac measuring approximately 1.15 cm × 2.39 cm.

**Figure 2:**
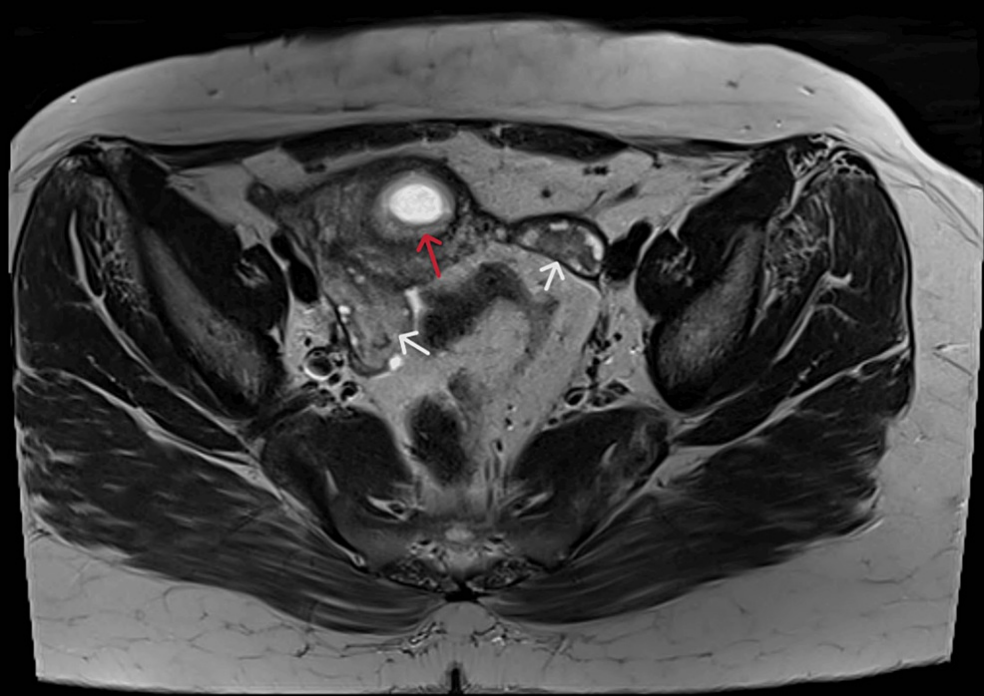
T2-weighted axial magnetic resonance imaging with the red arrow representing the gestational sac.

A 28-year-old patient, third gravida, with two living children, presented in our emergency labor ward with lower abdominal pain for eight hours and vaginal spotting for six hours with a positive urine pregnancy test and a transvaginal ultrasonography report suggestive of a cystic lesion in the anterior lower uterine segment with thinning of myometrium in that region suggesting a CSEP and a beta-human chorionic gonadotropin (β-hCG) level of 9,439 mIU/mL. The patient had a history of two cesarean deliveries in the past due to premature rupture of membranes with cephalopelvic disproportion in her first pregnancy, and the second was an elective cesarean because of a thin scar of a previous cesarean section. Her last cesarean section was two years before the presentation. Her menstrual cycles were regular, and she had no significant past, personal, or family history. She was well built on general examination with a body mass index of 25.6 kg/m^2^. Her vital parameters were stable, and on per abdominal examination, a skin scar of Pfannenstiel’s incision was seen. Her abdomen was soft and non-tender on palpation. On per speculum examination, dark red blood was visualized in the posterior fornix. A gentle per vaginal examination was done with findings conclusive of a bulky uterus corresponding to six to eight weeks of gestation, anteverted with bilateral fornices that were free and non-tender with no evidence of cervical motion tenderness. All relevant biochemical, serological, and hematological laboratory investigations were sent and serum β-hCG repeated on the day of admission was 27,133 mIU/mL. Ultrasonography was repeated at our institute and was suggestive of live cesarean scar ectopic gestation corresponding to seven weeks and six days gestation with a residual myometrial thickness of 2.3 mm which was confirmed using MRI (Figures 1, 2).

The patient was counseled regarding her high-risk condition which involved the possibility of massive hemorrhage, possible uterine scar rupture, and hysterectomy if needed. The patient was also explained different modes of management in detail which included medical management with systemic methotrexate, surgical management with hysteroscopic surgical evacuation/evacuation under ultrasound guidance, laparoscopy-assisted operative hysteroscopy management, laparoscopic assistance with excision and repair of the surgical scar, wedge resection, intragestational injection of methotrexate or KCl, transcervical balloon catheterization, and combined medical and surgical management along with a comprehensive explanation of associated benefits and risks. The patient refused surgical management and insisted on medical management despite counseling and was willing to stay in the hospital for as many days as needed clinically and agreed to surgical intervention anytime if required. High-risk informed consent was taken and blood was reserved. The emergency need for an operation theater was communicated to the staff and anesthesia team.

**Figure 3:**
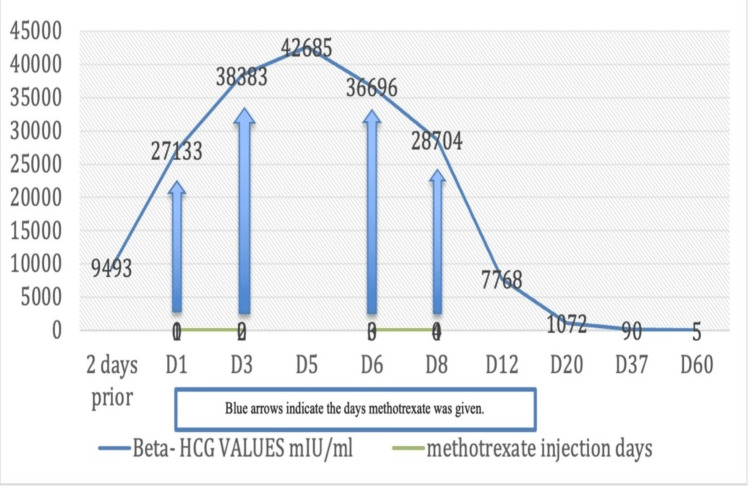
Graphical representation of β-hCG values on various treatment days with blue arrows indicating when methotrexate was given. β-hCG = beta-human chorionic gonadotropin; D = day

As the patient was hemodynamically stable and had normal baseline laboratory results, a multiple-dose regimen with systemic methotrexate (1 mg/kg) and leucovorin (0.1 mg/kg) rescue was initiated, despite the elevated baseline β-hCG value. A total of four doses of injection methotrexate 50 mg was followed by four doses of injection leucovorin 5 mg. On the first day of admission, the patient was given the first dose of injection methotrexate 50 mg deep intramuscularly. On the third day, a second dose of injection methotrexate 50 mg was given deep intramuscularly. As the β-hCG levels remained high on the fourth day of admission, the decision was made to administer an oral dose of 200 mg of mifepristone, considering the further increase of β-hCG levels to 38,383 mIU/mL. On the fifth day of admission, the patient had a single episode of fever of 100.1°F (37.8°C) due to which the third dose of injection methotrexate 50 mg was withheld and antibiotics were given. Her hemogram and β-hCG levels were serially monitored on different days of treatment (Tables 1, 2). On the sixth day of admission for the first time, there was a decline in β-hCG value to 36,696 mIU/mL, and a repeat ultrasound revealed single cesarean scar gestation with no fetal cardiac activity. She was then given a third dose of injection methotrexate 50 mg intramuscularly. On the eighth day, the last dose or fourth dose of injection methotrexate 50 mg was given deep intramuscularly. After each injection of methotrexate 50 mg, a 5 mg dose of leucovorin was administered intramuscularly 24 hours later. On the thirteenth day of admission, β-hCG levels dropped to 7,768 mIU/mL after which the patient decided to go home with all preparedness and to reach the hospital as soon as she experienced bleeding or other complications and was asked to follow up on outpatient department basis. Figure 3 displays a graphical representation depicting the changes in β-hCG levels over various treatment days, outlining both the increase and decrease in values.

**Table 1:** Representation of hemogram values on different days of admission.

Parameters	Day 1	Day 3	Day 5	Day 6	Day 8	Day 13
Hemoglobin (g/dL)	11.9	11.9	12.5	11.9	12	12.1
Total leukocyte count (/µL)	11,900	9,760	10,100	6,400	7,200	7,500
Platelet count (/µL)	299,000	285,000	304,000	244,000	244,000	236,000
Neutrophils (%)	82	72	88	80	57	72
Absolute neutrophil count (/µL)	9,758	7,027	8,888	5,120	4,101	5,400

**Table 2:** Tabular representation of βhCG on various days of admission or treatment. β-hCG = beta-human chorionic gonadotropin; D = day

Days of admission/treatment	β-hCG values
2 days prior to treatment	9,439 mIU/mL
D1	27,133 mIU/mL
D3	38,383 mIU/mL
D4	Tab. mifepristone 200 mg given
D5	42,685 mIU/mL
D6	36,696 mIU/mL
D8	28,704 mIU/mL
D13	7,768 mIU/mL
D20	1,072 mIU/mL
D37	90.7 mIU/mL
D60	5.18 mIU/mL

The patient followed up with a β-hCG report after one week of discharge which was 1,072 mIU/mL. The patient was followed up regularly and her laboratory investigations were within normal limits. After two months, her β-hCG dropped to 5.18 mIU/mL (non-pregnant range) and her ultrasound was suggestive of a resolved scar ectopic or complete abortion.

## Discussion

The term cesarean scar ectopic pregnancy delineates a variant of ectopic pregnancy wherein the gestational sac implants entirely or partially beyond the confines of the uterine cavity, specifically within the site of a prior cesarean scar situated in the lower uterine segment [1].

Maternal age >35 years, gravidity surpassing three, more than two induced abortions, an interval of fewer than five years between her current pregnancy and the last cesarean section, and a history of a cesarean section conducted at a rural community hospital have been identified as potential independent risk factors for this condition [2].

The diagnosis relies upon the evaluation of symptoms, clinical manifestations, a history of prior scarring, serum β-hCG levels, and transvaginal ultrasonography [3]. Ultrasound is the initial imaging test of choice for diagnosis of CSEP with a sensitivity of 86.4% [4]. To confirm our patient’s ultrasound findings an MRI was done as it provides superior soft tissue characterization and anatomical information that allowed us to consider conservative management. Diagnosis depends on symptoms, clinical manifestation, a history of previous scars, and serum β-hCG level transvaginal sonography.

It is recommended that systemic methotrexate should be the first line of management for ectopic pregnancy if the woman can follow up regularly and if she fulfills the criteria, which include women with no significant pain, unruptured ectopic mass <35 mm with no visible heartbeat present on the scan, β-hCG <5,000 mIU/mL, and absence of an intrauterine pregnancy as per National Institute for Health and Care Excellence guidelines [5].

Our patient was ideally not a good candidate for medical management, but she denied surgical intervention until necessary and was willing to attend for follow-up [5]. The treatment course depends on several variables, such as gestational age, hemodynamic stability, the accessibility of endoscopic knowledge, potential fertility concerns, and the viability of routine follow-up evaluations using imaging and serological modalities [1]. The primary objective of the management was to mitigate potential complications, including excessive hemorrhage, uterine rupture, and disseminated intravascular coagulation. This approach aimed to preserve the woman’s overall health and quality of life [1].

Our main modality of management was medical management as our patient was vitally stable with a single live cesarean scar ectopic gestation of fewer than eight weeks with mild per vaginal spotting. As she was reluctant to undergo surgical management, we initiated the multi-dose regimen of systemic methotrexate with folinic acid rescue after counseling her regarding all the benefits and risks of the same and that surgical intervention may be required in case of failure.

In the 1960s, Methotrexate was initially used in the management of confirmed ectopic pregnancies [6]. In systemic therapy involving methotrexate injections, both single and multiple-dose regimens have been applied. The established protocol for a single-dose regimen involves the administration of intramuscular methotrexate at a dosage of 50 mg/m^2^. In the multi-dose protocol, methotrexate injection is administered at a dosage of 1 mg/kg intramuscularly, alternating with folinic acid given at a dose of 0.1 mg/kg [7].

Methotrexate functions as a folic acid receptor antagonist, competitively inhibiting the enzyme dihydrofolate reductase, and thereby impeding the conversion of dihydrofolate to tetrahydrofolate ultimately interfering with trophoblastic cell replication [8]. Leucovorin, an active metabolite of folic acid and a crucial coenzyme in nucleic acid synthesis, is utilized to selectively rescue cells from the detrimental effects of methotrexate. Additionally, it facilitates the continuation of nucleic acid synthesis even in the presence of methotrexate, thereby preventing toxicity [9,10]. Even when administered at low doses, methotrexate is not devoid of side effects. The primary adverse effects typically involve symptoms such as nausea, vomiting, mucosal ulcers, a decrease in appetite, and skin rashes. Furthermore, elevated doses may result in additional severe and potentially life-threatening side effects, including alopecia, fatigue, infections, fever, gastrointestinal bleeding, pancreatitis, bone marrow suppression, renal failure, and malignancies such as lymphoproliferative disorders [11,12].

As her baseline β-hCG was still high (>15% rise) we decided to add an additional drug, mifepristone, to provide a synergistic and embryotoxic effect to reduce her β-hCG levels with the added advantage of no drug interaction [13]. Additional drugs may also be used along with medical management with systemic methotrexate such as mifepristone or potassium chloride. In a meta-analysis conducted by Su et al., both methotrexate and mifepristone were utilized, and the findings indicated that the combination therapy of mifepristone with methotrexate demonstrates superior curative effects. This combination approach was associated with improvements in the cure rate, reduction of β-hCG levels, diminished mass, and alleviation of symptoms such as abdominal pain and bleeding, all achieved without exacerbating toxic side effects [14]. Mifepristone, characterized as a steroidal anti-progesterone, functions by inhibiting the release of progesterone, ultimately inducing cell degeneration. The concomitant use of methotrexate enhances the dissolution of trophoblastic cells at a faster rate than systemic methotrexate alone [15]. In a small-scale study, the comparative efficacy of the combined regimen of mifepristone and methotrexate was assessed against the administration of a solitary dose of methotrexate at 50 mg/m^2^. The study findings indicated that the combined approach of mifepristone and methotrexate resulted in a decreased risk of treatment failure in the medical management of ectopic pregnancy [16]. In several case series, uterine artery embolization has also been combined with the use of local or systemic methotrexate to prevent massive hemorrhage [17]. In another study, cases of interstitial and cervical pregnancies were successfully managed conservatively with methotrexate and mifepristone with additional minimally invasive interventions [18]. Our patient was closely monitored throughout admission and there were no signs of neutropenia or any other abnormality in the laboratory reports despite suffering one episode of fever of 100.1°F.

Medical management of ectopic pregnancy requires close follow-up with provisions for a combined surgical approach, either an elective or emergency in case of heavy bleeding. Surgical interventions can involve hysteroscopic suction evacuation and curettage, laparoscopic or open removal of scar tissue in conjunction with pregnancy, and the use of hemostatic measures such as a double balloon catheter for tamponade and uterine artery embolization [14]. In a case series by Ash et al., a combined approach of hysteroscopy and laparoscopy was employed when ultrasound indicated a residual anterior myometrial thickness of less than 3 mm [3]. While there are several treatment options available for this condition, there is no universally recommended treatment of choice. In our patient’s case, even though many guidelines discourage medical management and lean toward surgical intervention, we believe further research is necessary, especially regarding combined medical management. Therefore, it is not accurate to assert that a singular, specific treatment can be universally applied based on various guidelines. Treatment should be tailored to each patient’s individual needs. Opting for medical management despite high β-hCG levels is a cost-effective alternative that avoids surgery and its associated complications, especially when the woman has completed her family and a permanent method of contraception can be provided Additionally, the recurrence rate of CSEP at the same scar defect site is less. Drawbacks of medical management include a lengthier treatment duration, uncertainty regarding the outcome, and the need for repetitive monitoring of β-hCG levels and other hematological investigations. Close supervision of the patient is essential due to the potential risk of catastrophic bleeding.

## Conclusions

CSEP represents a dangerous and intricate disorder that presents a diagnostic challenge, exhibiting a rising incidence in recent years concurrent with the escalating global prevalence of cesarean sections. It is imperative for clinicians and radiologists overseeing individuals with predisposing factors for scar ectopic pregnancy to uphold a heightened level of vigilance during imaging procedures and subsequent monitoring. The failure to promptly diagnose and initiate appropriate management can result in uterine rupture, extensive hemorrhage, and maternal mortality. Treatment should be highly individualized depending on the case.

Medical management with a combination of mifepristone and systemic methotrexate may be used in CSEP patients for better curative effects. However, its applicability depends on the specific case and patient compliance and necessitates further research for validation. Following a clinical evaluation, women should be granted the autonomy to make decisions about their healthcare. If a woman is contending with financial or social constraints, or if surgery is not her initial preference, the possibility of opting for medical management should be offered.
